# Dysfunctional Decidual CD4^+^T Cells Induce Recurrent Pregnancy Loss via Palmitoylation‐Dependent Tim‐3 Lysosomal Sorting and Degradation

**DOI:** 10.1002/advs.202500971

**Published:** 2025-07-11

**Authors:** Liyuan Cui, Xinhang Meng, Yujie Luo, Jinfeng Qian, Fengrun Sun, Mingke Qiu, Songcun Wang

**Affiliations:** ^1^ Laboratory for Reproductive Immunology Hospital of Obstetrics and Gynecology Fudan University Shanghai Medical College Shanghai 200092 China; ^2^ Department of Interventional Vascular Surgery Xinhua Hospital Shanghai JiaoTong University, School of Medicine Shanghai 200090 China

**Keywords:** decidual CD4^+^T cells, maternal‐fetal tolerance, palmitoylation, recurrent pregnancy loss, Tim‐3

## Abstract

Palmitoylation is a fully reversible post‐translational modification that regulates the functions of various proteins. While its dysregulation has been implicated in numerous pathological processes, there has been scarce researches on palmitoylation changes in proteins associated with recurrent pregnancy loss (RPL). Utilizing palmitoylation proteomics, 3262 proteins are identified with increased palmitoylation and 1577 proteins with decreased palmitoylation in the decidual immune cells from RPL patients compared to those from normal early pregnancies. Reduced palmitoylation by 2‐bromopalmitate promoted decidual CD4^+^T (dCD4^+^T) cell tolerance and decrease the resorption rate of abortion‐prone mice, suggesting that elevated palmitoylation level may be associated with RPL. The bioinformatics, proteomic, functional and mouse model studies revealed that T cell immunoglobulin domain and mucin domain‐3 (Tim‐3) is palmitoylated by ZDHHC3 on cysteine 9 residue. This palmitoylated‐Tim‐3 subsequently bound to sortilin and was directed to lysosome for degradation, leading to decreased expression of Tim‐3 and the dysfunction of dCD4^+^T cells, ultimately resulting in fetal loss. We elucidated a pivotal role for dynamic palmitoylation in RPL and uncovered a novel mechanism governing Tim‐3 regulation. Given that Tim‐3 dysregulation is frequently linked to RPL, our findings may have important implications for understanding the etiology of RPL and developing targeted therapy of RPL.

## Graphical abstract

Dysfunctional decidual CD4^+^T cells induce recurrent pregnancy loss via palmitoylation‐dependent Tim‐3 lysosomal sorting and degradation. The integrated bioinformatics, proteomic, functional and model studies elucidate that Tim‐3 is palmitoylated by ZDHHC3 at Cys9. Sortilin bound to and directed palmitoylated Tim‐3 toward lysosome degradation, resulting in diminished Tim‐3 expression and the subsequent dysfunction of dCD4^+^T cells, which contributes to fetal loss. Inhibition of palmitoylation by 2BP increases Tim‐3 expression on dCD4^+^T cells, promotes dCD4^+^T cell tolerance and decreases the embryo resorption rate of AP mice. This schematic clarifies a central role for dynamic palmitoylation in RPL, and uncovers a regulatory axis governing Tim‐3 homeostasis.

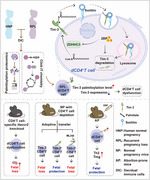

## 1 Introduction

Recurrent pregnancy loss (RPL) is defined as the spontaneous miscarriage of two or more pregnancies before the fetus reaches viability.^[^ ^1^ ^]^ The upper limit for the gestational age at which an abortion is considered varies by country or region. The average incidence of RPL among women who achieve pregnancy ranges from 1% to 4%, reflecting different definition of miscarriage.^[^ ^2^, ^3^ ^]^ RPL is considered as a disorder that necessitating medical intervention due to its profound impact on women's physical and psychological health. In addition, women with a history of RPL are confronted with an increased risk of subsequent miscarriage and adverse perinatal outcomes, including preterm labor and placental abruption.^[^ ^4^ ^]^ Consequently, delving into the underlying mechanisms of RPL and pinpointing therapeutic targets could help improve the prognosis of RPL patients.

Although parental or embryonic chromosomal anomalies, uterine anatomic defects, endocrine disorders, infectious factors, and antiphospholipid syndrome are recognized as frequent causes of RPL, the etiology remains elusive in nearly 50% of RPL cases.^[^ ^5^ ^]^ In mammals, fertilization occurs within the female reproductive tract, the fetus matures within the uterus, and finally, the mother delivers the baby. The fetus, carrying a mix of maternal and paternal genetic material, presents a physiological challenge as it introduces foreign antigens to the mother's body, and often repeatedly. Despite being immunologically cognizant of these foreign fetal alloantigens, pregnant women do not mount a rejection response. During pregnancy, the maternal immune system is activated to safeguard against infections and meanwhile ensure the fetus is not rejected. Significant immunological changes occur in the uterus, particularly at the implantation site, where immune tolerance to fetal antigens is induced to allow the pregnancy to progress.^[^ ^6^ ^]^ Consequently, maternal‐fetal immune dysregulation has been hypothesized as a potential contributor in cases of unexplained RPL.^[^ ^7^ ^]^

The modulation of the maternal decidual CD4^+^T (dCD4^+^T) cells response to fetal antigens is thought to be a vital component of maternal‐fetal tolerance during pregnancy. Upon encountering antigens presented by antigen presenting cells or driven by a set of cytokines, naive CD4^+^ T cells are able to differentiate into distinct subsets, including T helper 1 (Th1), Th2, Th17, and regulatory T cells (Tregs).^[^ ^8^ ^]^ Though pro‐inflammatory microenvironment is needed for the growth and invasion of trophoblasts.^[^ ^9^, ^10^ ^]^ Both an increased Th1/Th2 ratio and a decreased Treg/Th17 ratio have deleterious effects on pregnancy.^[^ ^11^, ^12^ ^]^ For many years, Treg expansion and a polarization toward Th2 bias in the maternal immune response have long been considered as the main mechanisms of maternal tolerance toward the fetus.^[^ ^13^ ^]^ We have previously shown that immune checkpoints T cell immunoglobulin domain and mucin domain‐3 (Tim‐3), programmed cell death protein 1 (PD‐1) and cytotoxic T‐lymphocyte‐associated protein 4 (CTLA‐4) are important for the function of dCD4^+^T cells. Treatment with these immune receptor‐blocking antibodies caused greater susceptibility to fetal loss with altered cytokine profiles by dCD4^+^T cells.^[^ ^14^, ^15^ ^]^ While the dysregulation of Tim‐3 in dCD4^+^T cells has been linked to RPL,^[^ ^16^ ^]^ the mechanisms underlying its abnormal expression remain elusive.

Post‐translational modifications, particularly palmitoylation, are emerging as pivotal regulators of immune receptor stability and function. Palmitoylation is a process of attaching a 16‐carbon palmitoyl group to substrate cysteine residues through thioester bonds.^[^ ^17^ ^]^ This post‐translational modification is catalyzed by a family of cysteine‐rich zinc finger proteins containing a conserved aspartic acid‐histidine‐histidine‐cysteine motif (ZDHHC), and it is reversed by acyl protein thioesterases.^[^ ^18^, ^19^ ^]^ The palmitoylation status can influence protein structure and stability, vesicle trafficking and membrane anchoring.^[^ ^20^ ^]^ Numerous immune receptors and effectors undergo palmitoylation that finely regulates their dynamic expression.^[^ ^21^ ^]^ A notable example is the degradation inhibition of programmed cell death ligand 1 (PD‐L1) undergoes palmitoylation.^[^ ^22^, ^23^ ^]^ Palmitoylation plays a crucial role in various aspects of tumor cell proliferation, metastasis and apoptosis by disturbing antitumor immunity in the tumor microenvironment.^[^ ^24^ ^]^ Enhancement of T‐cell immune responses against tumor were observed when the palmitoylation of PD‐L1 was inhibited.^[^ ^25^ ^]^ While palmitoylation of Tim‐3 promoted immune exhaustion and restrains antitumor immunity.^[^ ^26^ ^]^ Targeting specific palmitoylases or depalmitoylases represents a promising therapeutic strategy in antitumor therapeutics.^[^ ^20^, ^27^ ^]^

Due to the high proliferation, invasive properties, and the capacity to escape from immune attack, the placenta is regarded as a physiological metastasis or pseudo‐malignant type of tissue. As the embryo breaks through the endometrial epithelial basement membrane and invades the endometrial stroma, lymphocytes gather densely around the embryo. This scenario is analogous to the infiltration of lymphocytes around cancer cells when they penetrate the epithelial basement membrane and invade the stroma.^[^ ^6^, ^28^ ^]^ Despite these parallels, there are limited studies on palmitoylation changes in proteins linked to RPL. However, the regulatory effect of palmitoylation on tumor immunity provided us with great research inspiration on maternal‐fetal tolerance.

Given the parallels between immune tolerance in pregnancy and tumor microenvironment,^[^ ^6^ ^]^ we hypothesized that palmitoylation might similarly govern Tim‐3 expression in dCD4^+^T cells. Here, we combined palmitoyl‐proteomics and functional assays to identify Tim‐3 as a key target of palmitoylation in RPL, and further elucidated how this modification drove its degradation via the sortilin‐lysosome pathway, ultimately compromising maternal‐fetal tolerance.

## 2 Results

### 2.1 Palmitoylation Affected dCD4^+^T Cell Function during Pregnancy

**Figure 1:**
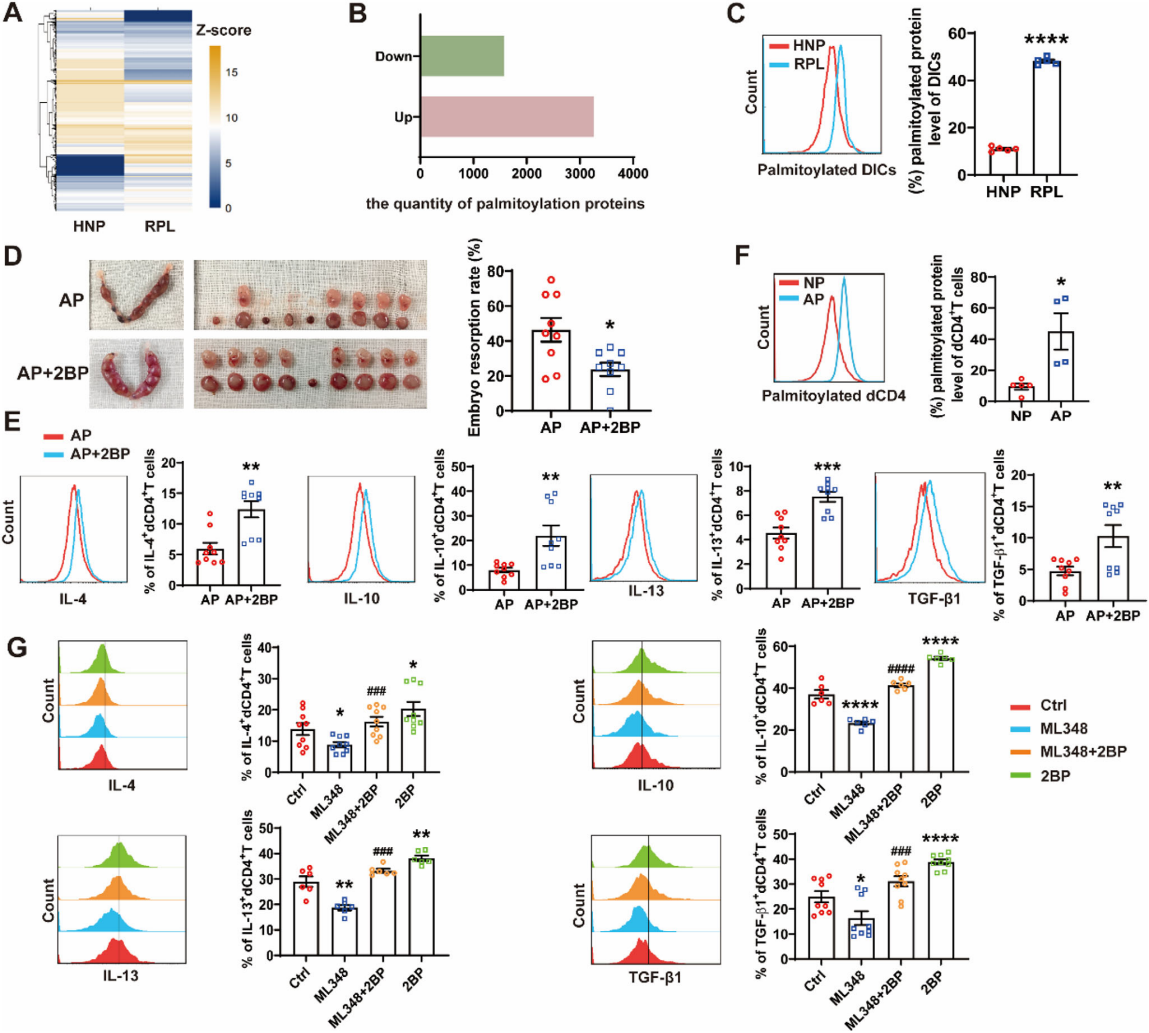
Palmitoylation affected dCD4^+^T cell function during pregnancy. A) and B) Hierarchical clustering heatmap of palmitoylated protein sites A) and quantification of palmitoylated proteins B) in decidual immune cells (DICs) between human normal pregnancies (HNP) and recurrent pregnancy loss patients (RPL). C) Comparative palmitoylated protein assay of DICs between HNP (n = 5) and RPL (n = 5). D) Establishment of abortion‐prone models (AP) by mating eight‐week‐old CBA/J females with DBA/2 males. The representative images of uterus and the percentage of fetal resorption of pregnant CBA/J females treated with (n = 9) or without 2BP (n = 9). E) Flow cytometric analysis and quantification of cytokine expression frequency in dCD4^+^T cells of pregnant CBA/J females following treatment with or without 2BP. F) Induction of normal pregnancy (NP) by mating eight‐week‐old CBA/J females with BALB/c males. Comparative palmitoylated protein assay of dCD4^+^T cells between NP (n = 5) and AP (n = 4). G) Flow cytometric analysis and quantification of cytokine expression frequency in dCD4^+^T cells from HNP following treatment with or without 10 µM ML348 and/or 50 µM 2BP for 48 h. Images are representative of three independent experiments. Data represent the mean ± standard error of the mean (SEM). *p < 0.05, **p < 0.01, ***p < 0.001, ****p < 0.0001, indicate statistical significance compared to the control (Ctrl) group. ###*p* < 0.001, ####*p* < 0.0001, compared to the ML348 group.

To investigate the role of palmitoylation in the maternal‐fetal tolerance within the context of RPL, we employed a proteomic approach focused on palmitoylation.^[^ ^29^ ^]^ **Figure** **1**A illustrates a heatmap depicting the comparative analysis of palmitoylated protein sites in decidual immune cells (DICs) derived from both human normal pregnancies (HNP) and RPL patients. Our analysis revealed a significant alteration in palmitoylation patterns, with 3262 proteins exhibiting increased palmitoylation level and 1577 proteins showing decreased palmitoylation level in RPL patients compared to HNP (Figure 1B). We further confirmed a higher abundance of palmitoylated proteins in DICs from RPL patients compared to HNP (Figure 1C). Among these immune cells, CD4^+^T cells demonstrated the most significant upregulation of palmitoylated proteins (Figure S1A, Supporting Information). The general palmitoylation inhibitor, 2‐bromopalmitate (2BP), decreased the embryo resorption rate but increased the placenta weight of abortion‐prone (AP) mice (Figure 1D; Figure S1B, Supporting Information), further suggesting a potential link between elevated palmitoylation and the pathogenesis of RPL.

In addition, 2BP was found to upregulate the expression of Th2 and Treg‐associated cytokines of dCD4^+^T cells from AP mice, including interleukin (IL)‐4, IL‐10, IL‐13 and transforming growth factor (TGF)‐β1 (Figure 1E). The proportion of palmitoylated proteins in dCD4^+^T cells from AP mice exceeded that observed in normal pregnancy (NP) mice, as shown in Figure 1F. Enhanced palmitoylation by ML348, a selective inhibitor of acyl protein thioesterase 1, suppressed Th2 and Treg‐associated cytokine production of human dCD4^+^T cells, while 2BP reserved this inhibition (Figure 1G). Collectively, these results underscored the influence of palmitoylation on dCD4^+^T cell function during pregnancy.

### 2.2 ZDHHC3 Palmitoylated Tim‐3 on Cys9 During Pregnancy

**Figure 2:**
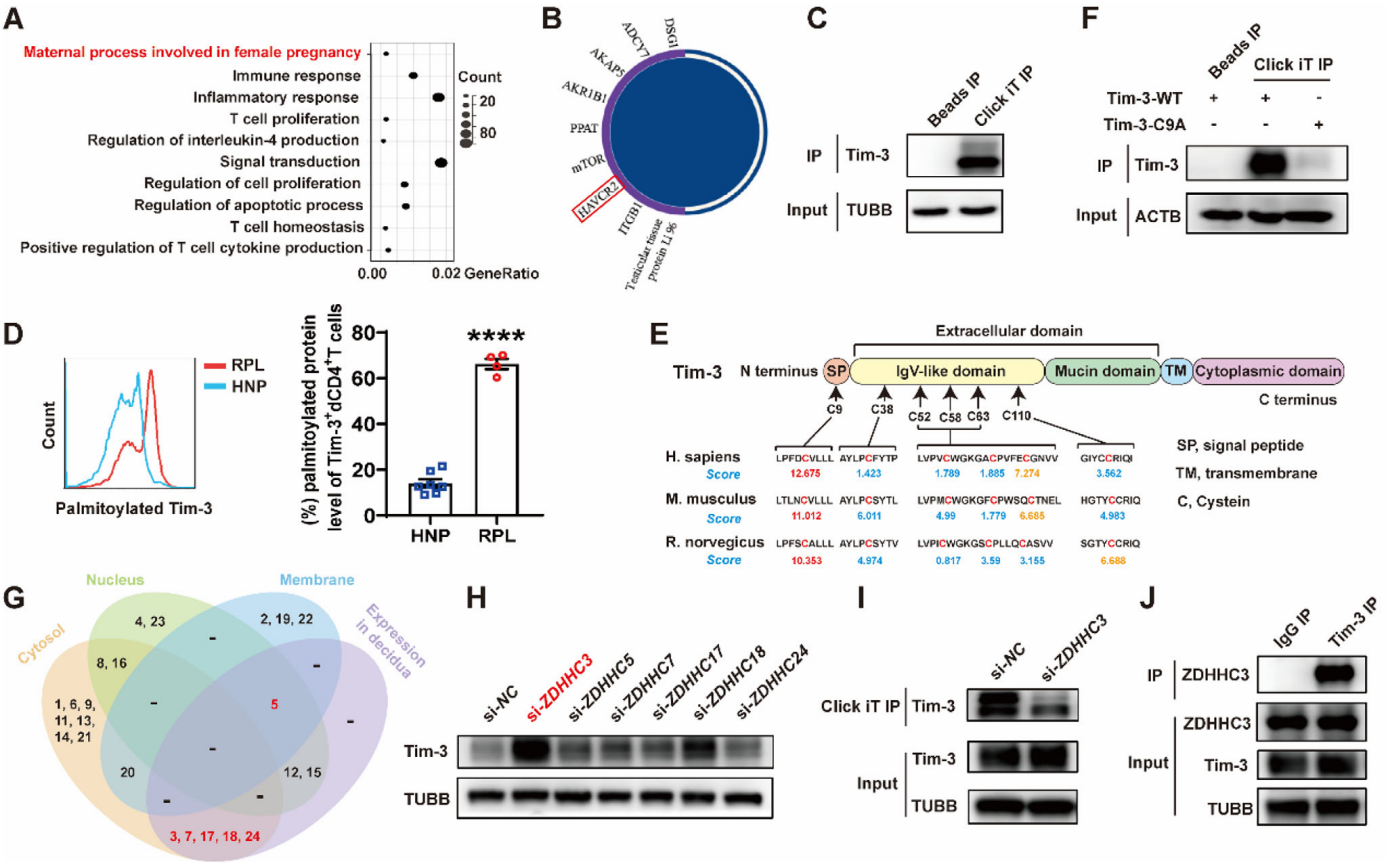
ZDHHC3 palmitoylated Tim‐3 on Cys9 during pregnancy. A) Gene Ontology term enrichment analysis highlighting differentially palmitoylated proteins pathways between HNP and RPL. B) Maternal processes involved in female pregnancy‐related differentially palmitoylated proteins between HNP and RPL. C) Click‐iT reaction for the detection of Tim‐3 palmitoylation. IP, immunoprecipitation. D) Comparative palmitoylated protein assay of Tim‐3^+^dCD4^+^T cells between HNP (n = 7) and RPL (n = 4). E) Alignment of protein sequences of Tim‐3 across various species revealed cysteine conservation. Six cysteine residues were predicted as palmitoylation sites of Tim‐3 in CSS‐palm 4.0 and Cys9 got the highest score. F) Quantification of palmitoylation levels of Tim‐3‐WT and C9A mutant (Tim‐3‐C9A) measured by Click‐iT assay. G) Venn diagram showing the expression patterns of different ZDHHCs isoforms in decidua, including both expression level and subcellular distribution. H) Expression of Tim‐3 in HEK293T cells following transfection with siRNAs targeting different ZDHHCs. I) Tim‐3 palmitoylation level in si‐*ZDHHC3* transfected HEK293T cells, as determined by Click‐iT assay. J) IP analysis confirming the interaction between Tim‐3 and ZDHHC3. Images are representative of three individual experiments.

To elucidate the impact of palmitoylation on pregnancy outcomes, we conducted a Gene Ontology (GO) analysis to identify differentially palmitoylated proteins between HNP and RPL patients. The results indicated that differentially palmitoylated proteins were predominantly enriched in maternal process involved in female pregnancy, immune response, inflammatory response, and so on (**Figure** **2**A). Specifically, 9 differentially palmitoylated proteins were identified, which are related to maternal process involved in female pregnancy (Figure 2B). Among these, hepatitis A virus cellular receptor 2 (HAVCR2) (also known as Tim‐3) aroused our interest due to its involvement in inflammatory responses. Tim‐3 signaling is crucial for preserving immune homeostasis.^[^ ^30^ ^]^ Blockade of Tim‐3 has been shown to impair scavenging activity to dead and apoptotic cells from the uterus, leading to increased embryo resorption.^[^ ^31^ ^]^ Tim‐3 signal is also important in maternal‐fetal tolerance and trophoblast invasion.^[^ ^16^ ^]^ To verify whether Tim‐3 is the key palmitoylated protein regulating dCD4^+^T cell function, we validated its palmitoylation by Click‐iT assay firstly. As shown in Figure 2C, streptavidin pulldown and immunoblot using Tim‐3 antibody confirmed the palmitoylation of Tim‐3. Furthermore, the percent of palmitoylated Tim‐3 in dCD4^+^T cells from RPL patients was found to be higher than in those from HNP (Figure 2D).

To pinpoint the palmitoylation site of Tim‐3, we employed the motif‐based predictors CSS‐palm 4.0.^[^ ^32^ ^]^ Six conserved cysteine residues across the different species were predicted as potential palmitoylation sites of Tim‐3, with cysteine 9 residue (Cys9) within the signal peptide domain receiving the highest score (Figure 2E). Mutation of Cys9 to alanine nearly abolished the palmitoylation of Tim‐3, which confirmed the palmitoylation on Cys9 (Figure 2F).

In an effort to identify the predominant palmitoyltransferase(s) responsible for Tim‐3 palmitoylation during pregnancy, we applied a screening strategy that integrated protein expression analysis, subcellular localization analysis and loss‐of‐function test. Initially, we assessed the expression of all ZDHHC family members in human decidua based on the Human Protein Atlas.^[^ ^33^ ^]^ By excluding nuclear‐localized ZDHHCs that do not spatially overlap with Tim‐3, we identified candidate palmitoyltransferases for experimental validation, namely ZDHHC3, ZDHHC5, ZDHHC7, ZDHHC17, ZDHHC18 and ZDHHC24 (Figure 2G). Subsequently, specific small interfering RNAs (siRNAs) were used to respectively silence these genes, allowing loss‐of‐function screening. As shown in Figure 2H,I, knockdown of *ZDHHC3* significantly promoted Tim‐3 protein expression (Figure 2H) and suppressed Tim‐3 palmitoylation (Figure 2I). Co‐immunoprecipitation (IP) suggested a physical interaction between ZDHHC3 and Tim‐3 (Figure 2J). Consistent with this, ZDHHC3 was found to colocalize with Tim‐3 on CD4^+^T cells in decidual tissues from both HNP and RPL patients (Figure S2A, Supporting Information). And ZDHHC3 expression was higher in dCD4^+^T cells of RPL patients (Figure S2B, Supporting Information). In addition, enhancement of palmitoylation induced by ML348 or *ZDHHC3* overexpression significantly decreased Tim‐3 protein expression in Jurkat T cells, while ML348 or *ZDHHC3* overexpression had no effect on Tim‐3 expression level of the palmitoylation‐deficient Tim‐3‐C9A mutant (Figure S2C, Supporting Information). These results consistently suggested that ZDHHC3 is responsible for the palmitoylation of Tim‐3 on Cys9 during pregnancy.

### 2.3 Palmitoylation Regulated Lysosomal Degradation of Tim‐3 on dCD4^+^T Cells

**Figure 3:**
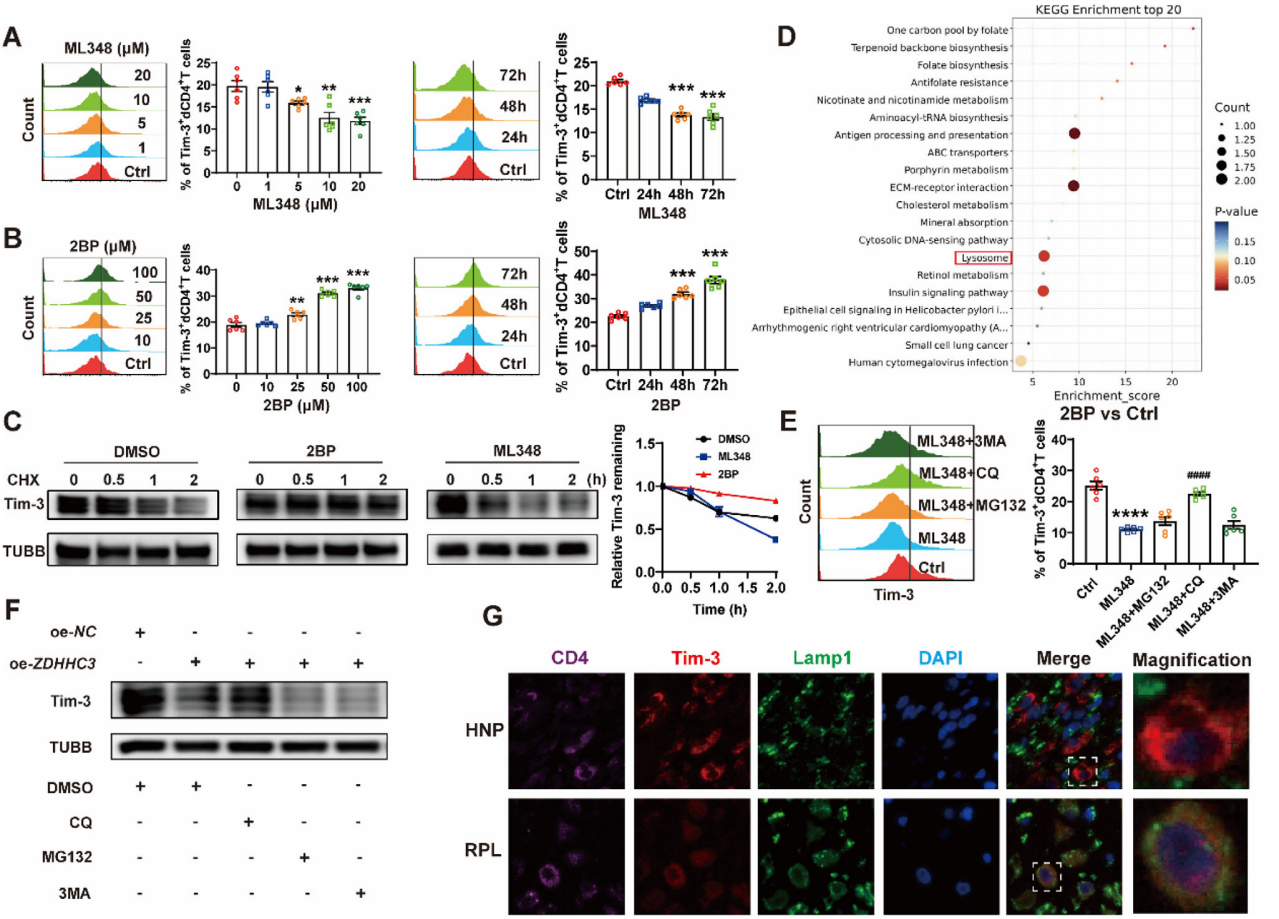
Palmitoylation regulated lysosomal degradation of Tim‐3 on dCD4^+^T cells. A) Flow cytometric quantification of Tim‐3 expression on dCD4^+^T cells treated with the indicated concentrations (0, 1, 5, 10, 20 µM) of ML348 for 48 h or with 10 µM ML348 for indicated durations (0, 24, 48, 72 h). B) Flow cytometric quantification of Tim‐3 expression on dCD4^+^T cells treated with the indicated concentrations (0, 10, 25, 50, 100 µM) of 2BP for 48 h or with 50 µM 2BP for indicated durations (0, 24, 48, 72 h). C) Degradation of Tim‐3 in 2BP (50 µM), ML348 (10 µM) or dimethyl sulfoxide (DMSO) treated HEK293T cells measured by CHX chase assay. Relative Tim‐3 protein levels, normalized to TUBB, were presented relative to the level at 0 h mark after CHX treatment. D) Kyoto Encyclopedia of Genes and Genomes (KEGG) analysis results of differentially expressed proteins between dCD4^+^T cells with and without 50 µM 2BP treatment by mass spectrometry. E) Flow cytometric quantification of Tim‐3 expression on dCD4^+^T cells treated with the 10 µM ML348 with or without indicated inhibitors targeting various degradation pathways (1 µM MG132, 20 µM CQ, 10 mM 3MA). F) Tim‐3 protein levels in HEK293T cells co‐transfected with *Tim ‐ 3 ‐ WT* plasmid and oe‐*ZDHHC3* in the presence or absence of indicated inhibitors of different degradation pathways. G) Representative images depicting the colocalization of Tim‐3 and Lamp1 in dCD4^+^T cells from paraffin sections of decidual tissue of HNP and RPL. Images are representative of three individual experiments. Data represent the mean ± SEM from three independent analyses. *p < 0.05, **p < 0.01, ***p < 0.001, ****p < 0.0001, compared to the Ctrl group (including 0 h), ####*p* < 0.0001, compared to the ML348 group.

Given that *ZDHHC3* knockdown significantly elevated Tim‐3 protein expression (Figure 2H), along with that Tim‐3 expression was downregulated, while ZDHHC3 expression increased in dCD4^+^T cells of RPL patients (Figure S2A, B, Supporting Information), we hypothesized palmitoylation may modulate dCD4^+^T cell function during pregnancy via regulating Tim‐3 expression. ML348 treatment on dCD4^+^T cells enhanced protein palmitoylation, resulting in dose‐ and time‐dependent decrease in Tim‐3 expression (**Figure** **3**A). Oppositely, inhibition of palmitoylation by 2BP led to an increase in Tim‐3 expression on dCD4^+^T cells (Figure 3B). Further cycloheximide (CHX)‐chase assay revealed that ML348‐induced enhancement of palmitoylation accelerated Tim‐3 degradation, while palmitoylation suppression by 2BP, Cys9 mutation, or si‐*ZDHHC3* stabilized Tim‐3 (Figure 3C; Figure S3A, Supporting Information).

To further investigate the regulatory mechanisms by which palmitoylation influences Tim‐3 stability, we performed a mass spectrometry‐based screen of differentially expressed proteins between dCD4^+^T cells with and without 2BP treatment. The Kyoto Encyclopedia of Genes and Genomes (KEGG) analysis revealed an enrichment of the differentially expressed proteins in lysosome related signaling pathway (Figure 3D). To confirm the lysosome‐dependent mechanism, we examined the effects of various small‐molecular inhibitors targeting distinct degradation pathways. As shown in Figure 3E; Figure S3B (Supporting Information), ML348‐induced Tim‐3 downregulation could be rescued by Cys9 mutant or chloroquine (CQ, a lysosome inhibitor), but not MG132 (a proteasome inhibitor) or 3‐Methyladenine (3MA, an autophagy inhibitor). CQ also reversed the effect of oe‐*ZDHHC3* on Tim‐3 expression (Figure 3F). 2BP treatment substantially decreased the colocalization between Tim‐3 and lysosome‐associated membrane protein 1 (Lamp1)‐labelled lysosomes, whereas ML348 increased their colocalization (Figure S3C, Supporting Information). Moreover, the colocalization of Tim‐3 and lysosomes on dCD4^+^T cells from RPL patients was more pronounced than on those from HNP (Figure 3G). Collectively, these results indicated that palmitoylation facilitated Tim‐3 degradation through the lysosomal pathway.

### 2.4 Palmitoylation Regulated Lysosomal Degradation of Tim‐3 via Sortilin

**Figure 4:**
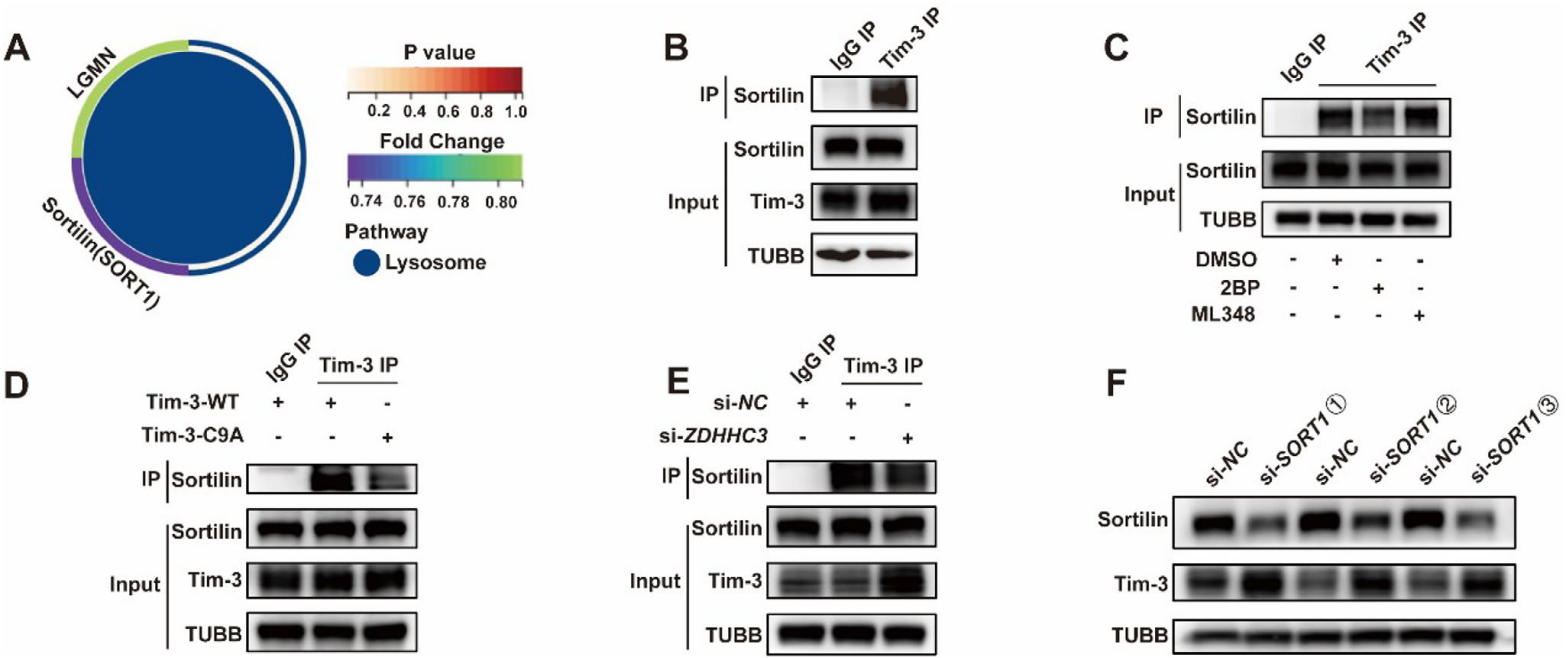
Palmitoylation regulated lysosomal degradation of Tim‐3 via sortilin. A) Lysosome‐related differentially expressed proteins under 50 µM 2BP treatment. B) IP analysis demonstrating the interaction of Tim‐3 and sortilin in HEK293T cells. C) IP analysis examining the interaction of Tim‐3 and sortilin in HEK293T cells treated with 50 µM 2BP or 10 µM ML348. D) IP analysis assessing the interaction between Tim‐3 or the palmitoylation‐deficient Tim‐3‐C9A mutant (Tim‐3‐C9A) and sortilin in HEK293T cells. E) IP analysis of the interaction between Tim‐3 and sortilin in HEK293T cells co‐transfected with *Tim ‐ 3 ‐ WT* plasmid and si‐*NC*/si‐*ZDHHC3*. F) Protein levels of Tim‐3 and sortilin in HEK293T cells co‐transfected with *Tim ‐ 3 ‐ WT* plasmid and various si‐*SORT1* constructs. Images are representative of three individual experiments.

Under 2BP treatment, we identified two lysosome‐related differentially expressed proteins (**Figure** **4**A). IP experiments revealed no interaction between Tim‐3 and Legumain (LGMN) (Figure S4A, Supporting Information), yet confirmed a significant interaction between Tim‐3 and sortilin (Figure 4B). Notably, diminished palmitoylation abrogated the interaction between Tim‐3 and sortilin (Figure 4C–E; Figure S4B, Supporting Information), while enhanced palmitoylation augmented this interaction (Figure 4C; Figure S4B, Supporting Information). Sortilin, a multifunctional receptor implicated in transport and sorting, is known to participate in various cellular processes, including intracellular sorting, enzyme and neuropeptide transport regulation, and cellular signaling.^[^ ^34^, ^35^ ^]^ Based on these findings, we hypothesized that sortilin might be involved in Tim‐3 lysosomal sorting for degradation. To validate this hypothesis, we knocked down *SORT1* (encodes sortilin) with specific siRNA. In line with our expectations, *SORT1* knockdown resulted in the increase in Tim‐3 protein levels (Figure 4F). In addition, we observed a heightened colocalization of Tim‐3 and sortilin in dCD4^+^T cells of RPL patients compared to those of HNP (Figure S4C, Supporting Information). Collectively, these results underscored the significance of palmitoylation in modulating the lysosomal degradation of Tim‐3 via sortilin.

### 2.5 Tim‐3 Induced Th2 and Treg Bias in dCD4^+^T Cells and Promoted the Maintenance of Normal Pregnancy

**Figure 5:**
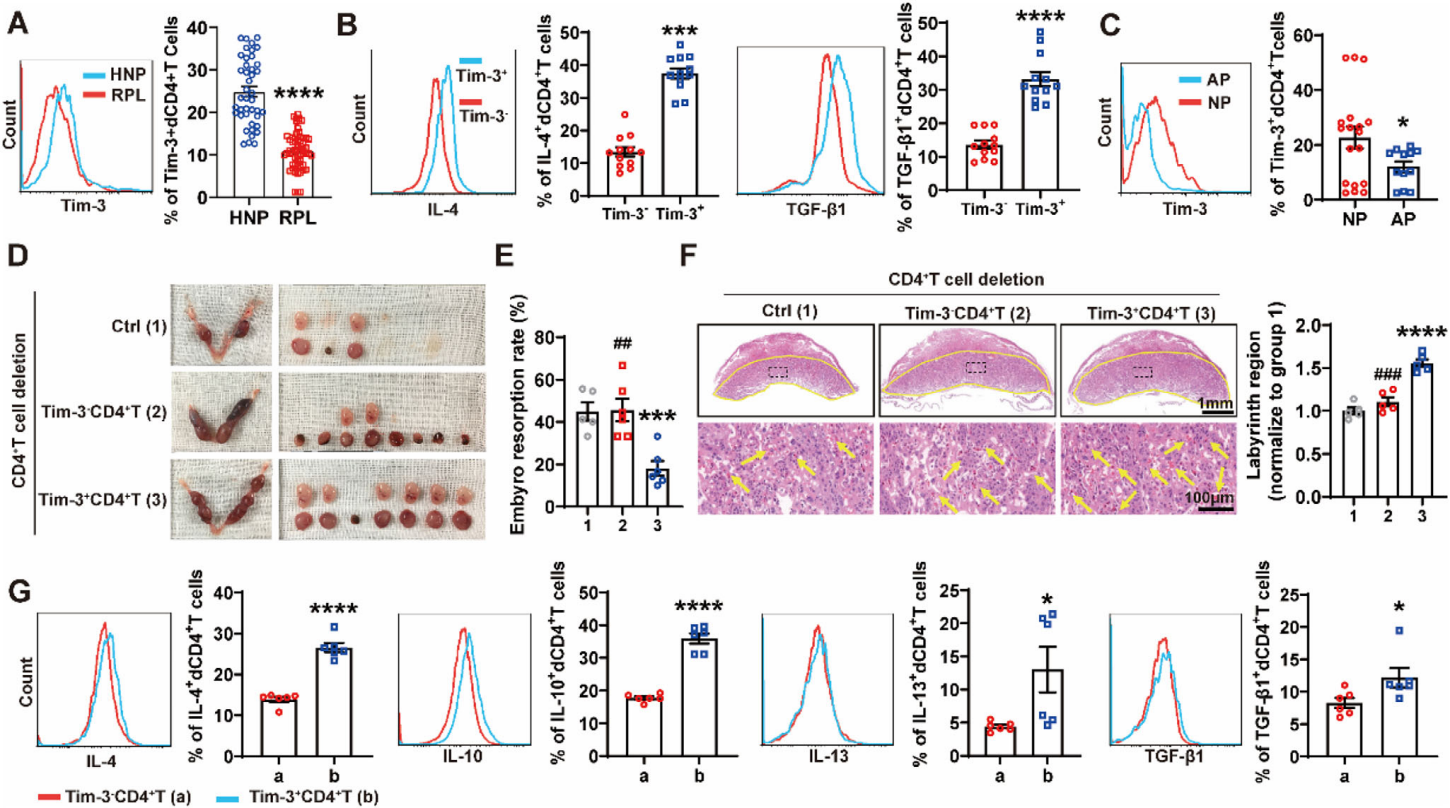
Adoptive transfer of Tim‐3^+^CD4^+^T cells relieved murine embryo resorption induced by CD4^+^T cell depletion. A) Flow cytometric analysis determining the frequency of Tim‐3 expressing cells in gated CD4^+^T cells of DICs from HNP (n = 42) and RPL patients (n = 63). B) Flow cytometric analysis and quantification of cytokine expression frequency by Tim‐3^+^dCD4^+^T cells or Tim‐3^−^dCD4^+^T cells from the first trimester of HNP (n = 12). C) Frequency of Tim‐3 expressing cells in gated CD4^+^T cells from DICs of normal pregnant (NP, n = 18) and abortion‐prone (AP, n = 12) mice. D and E) Representative uterus images (D) and embryo resorption rate (E) in pregnant CBA/J mice with CD4^+^T cell depletion (n = 5) and those receiving adoptive transfer of Tim‐3^+^CD4^+^T cells (n = 6) or Tim‐3^−^CD4^+^T cells (n = 6) at GD 13.5. F) HE staining of placental hemi‐sections at GD13.5 and quantitative analysis of labyrinthine zone area. Vascular branching in the labyrinthine zones was lined by endothelial‐like cells and contained erythrocytes. Histological analyses showed that endothelial cells in group (1) and (2) failed to form normal tubular vessel structures, instead forming clustered, unconnected, unstructured vessel‐like formations. G) Flow cytometric quantification of cytokine production by dCD4^+^T cells pregnant CBA/J females with CD4^+^T cell depletion that received adoptive transfer of Tim‐3^+^CD4^+^T cells or Tim‐3^−^CD4^+^T cells. Images are representative of three individual experiments. Data represent the mean ± SEM. *p < 0.05, ***p < 0.001, ****p < 0.0001; ##*p* < 0.01, ###*p* < 0.001, compared to the group (3).

We compared the frequency of Tim‐3^+^dCD4^+^T cells between HNP and RPL patients. Our findings revealed a reduced frequency of Tim‐3^+^dCD4^+^T cells in RPL patients compared to HNP (**Figure** **5**A), consistent with the results of immunofluorescence above. Additionally, Tim‐3^+^dCD4^+^T cells expressed more IL‐4 and TGF‐β1 than Tim‐3^−^dCD4^+^T cells (Figure 5B). A similar reduction of Tim‐3^+^dCD4^+^T cells was observed in AP mice relative to NP mice (Figure 5C). To clarify the possible role of the Tim‐3^+^CD4^+^T cells in murine pregnancy, we evaluated the pregnancy outcomes of NP mice with CD4^+^T depletion and subsequent adoptive transfer of either Tim‐3^+^CD4^+^T cells or Tim‐3^−^CD4^+^T cells. We found that adoptive transfer of Tim‐3^+^CD4^+^T cells, but not Tim‐3^−^CD4^+^T cells, significantly mitigated the embryo resorption (Figure 5D, E) and the placental dysplasia (Figure 5F) induced by CD4^+^T cell depletion. Moreover, the transfer of Tim‐3^+^CD4^+^T cells induced a Th2 and Treg bias in dCD4^+^T cells in CD4^+^T cell‐deleted pregnant mice. While the Tim‐3^−^CD4^+^T cell transferred group expressed reduced expression of IL‐4, IL‐13, IL‐10 and TGF‐β1 (Figure 5G). These results indicated that Tim‐3 induced Th2 and Treg bias in dCD4^+^T cells, thereby facilitating pregnancy preservation.

**Figure 6:**
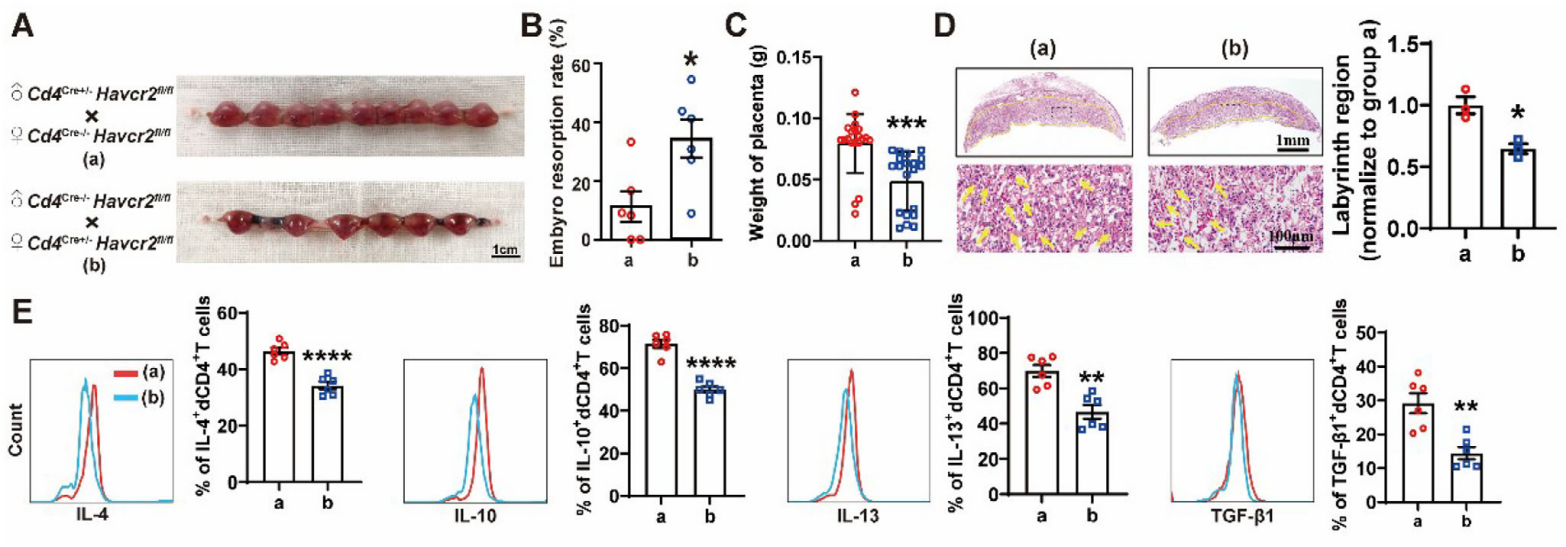
Conditional deletion of *HAVCR2* in CD4^+^T cells induced spontaneous abortion by impaired placental development and maternal‐fetal tolerance. A) Representative images of uterus from WT (♂*Cd4^Cre^* ^+/−^ *Havcr2* ^fl/fl^×♀*Cd4^Cre^* ^−/−^ *Havcr2* ^fl/fl^) and CD4^+^T cell‐specific Tim‐3 knockout (♂*Cd4^Cre‐^* ^/−^ *Havcr2* ^fl/fl^ × ♀*Cd4^Cre^* ^+/−^ *Havcr2* ^fl/fl^) pregnant mice. B)‐D) Analysis of embryo resorption rate B), placental weights C), HE staining of placental hemi‐sections and quantitative analysis of labyrinthine zone area D) of WT pregnant mice (n = 6) and CD4^+^T cell‐specific Tim‐3 knockout pregnant mice (n = 6) at GD13.5. Yellow arrows indicated erythrocytes. E) Flow cytometric quantification of cytokines expression by dCD4^+^T cells from pregnant females. Images are representative of three independent experiments. Data represent the mean ± SEM. *p < 0.05, **p < 0.01, ***p < 0.001, ****p < 0.0001.

To provide direct insight into the in vivo role of Tim‐3 on dCD4^+^T cells and pregnancy outcome, we generated CD4^+^T cell‐specific Tim‐3 knockout mouse models (**Figure** **6**A). In *Cd4^Cre^* ^+/−^ *Havcr2* ^fl/fl^ pregnant mice, we observed higher embryo resorption rates (Figure 6A, B), lower placenta weight (Figure 6C), and a striking defect in the formation of the labyrinth vasculature (Figure 6D). In addition, the specific knockout of Tim‐3 in CD4^+^T cells also resulted in impaired maternal‐fetal tolerance, as evidenced by decreased IL‐4, IL‐13, IL‐10 and TGF‐β1 expression in dCD4^+^T cells, evaluated by flow cytometry assays (Figure 6E). Collectively, these results demonstrated that specific knockout of Tim‐3 in CD4^+^T cells induced spontaneous abortion due to compromised placental development and maternal‐fetal tolerance.

### 2.6 Enhanced Palmitoylation of Tim‐3 Eliminated the Protective Effect of Tim‐3^+^CD4^+^T Cells on Murine Pregnancy

**Figure 7:**
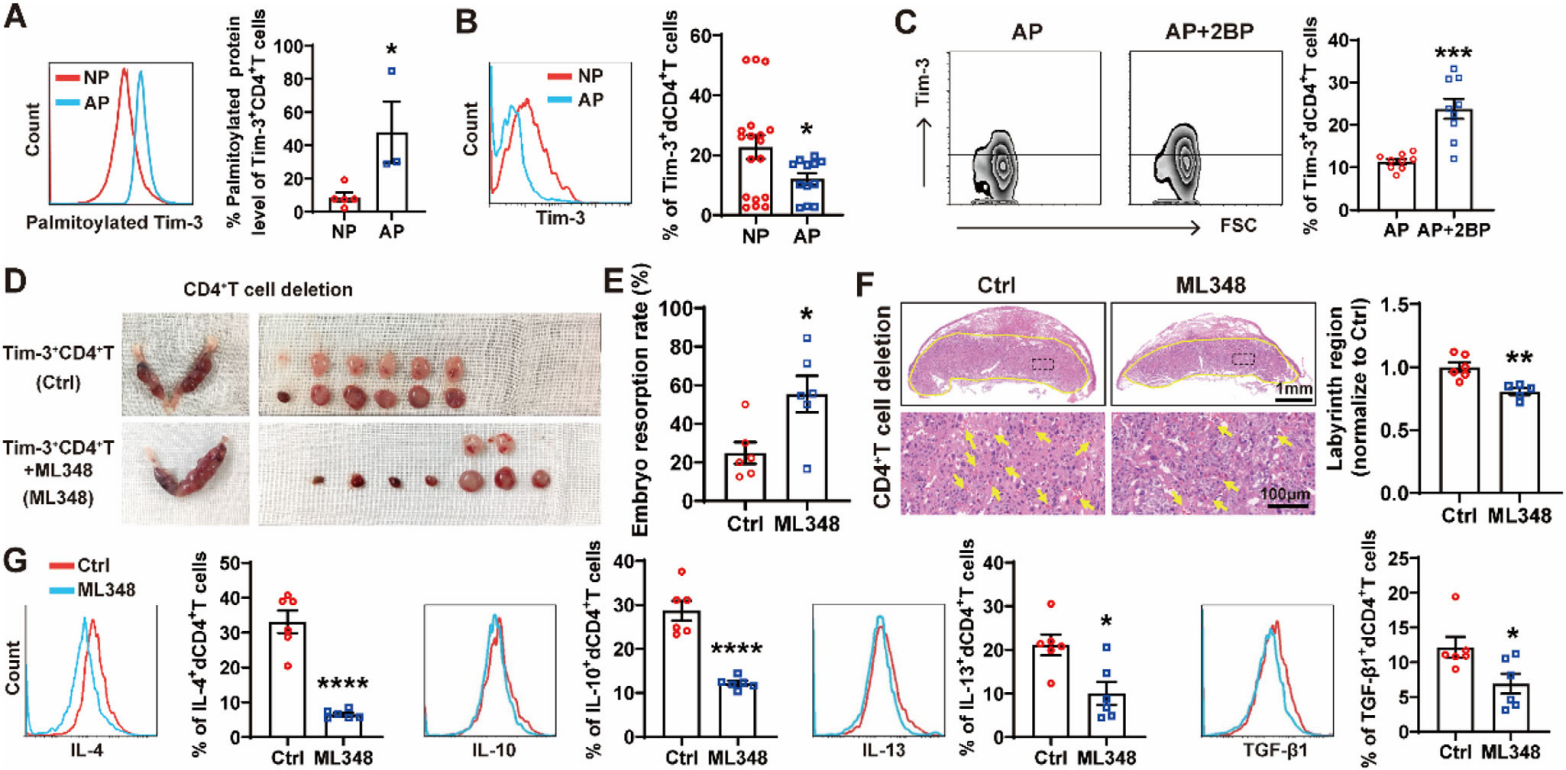
Enhanced palmitoylation of Tim‐3 eliminated the protective effect of Tim‐3^+^CD4^+^T cells on murine pregnancy. A) Palmitoylated protein assay of Tim‐3^+^dCD4^+^T cells between NP (n = 5) and AP (n = 3) mice. B) Flow cytometric determination of Tim‐3 expressing cells in gated CD4^+^T cells of DICs between NP (n = 18) and AP (n = 12) mice. C) Flow cytometric quantification of Tim‐3 expressing on dCD4^+^ T cells from AP mice with (n = 9) or without 2BP (n = 9) treatment. D) Representative images of uterus in pregnant CBA/J mice with CD4^+^T cell depletion that received adoptive transfer of Tim‐3^+^CD4^+^T cells (n = 6) or 10 µM ML348 pre‐treated Tim‐3^+^CD4^+^T cells (n = 6). E) and F) Embryo resorption rate (E), HE staining of placental hemi‐sections and quantitative analysis of labyrinthine zone area (F) of GD13.5 embryos. G) Flow cytometric quantification of cytokine production by dCD4^+^ T cells in pregnant CBA/J females with CD4^+^T cell depletion that received adoptive transfer of indicated Tim‐3^+^CD4^+^T cells. Images are representative of three individual experiments. Data represent the mean ± SEM. *p < 0.05, **p < 0.01, ***p < 0.001, ****p < 0.0001.

The percent of palmitoylated Tim‐3 in dCD4^+^T cells derived from AP mice was observed to be higher than that in those from NP mice (**Figure** **7**A). Additionally, ZDHHC3 was found to colocalize with Tim‐3 in decidual tissues from both NP and AP mice (Figure S5, Supporting Information). Concurrently, the frequency of Tim‐3^+^dCD4^+^T cells was lower in AP mice compared to NP mice (Figure 7B). Furthermore, the inhibition of palmitoylation by 2BP increased the expression of Tim‐3 in dCD4^+^T cells of AP mice (Figure 7C). These data suggested that enhanced palmitoylation of Tim‐3 was associated with the lower expression of Tim‐3, potentially contributing to the pathogenesis of miscarriage.

To delineate the putative influence of palmitoylation on Tim‐3^+^CD4^+^T cells in murine pregnancy, we conducted additional experiments to determine if augmenting palmitoylation could alter the pregnancy outcome in mice that received CD4^+^T cell deletion and adoptive transfer of Tim‐3^+^CD4^+^T cells. Our findings, depicted in Figure 7D–F, revealed that administration of ML348 counteracted the protective effect of Tim‐3^+^CD4^+^T cell adoptive transfer on CD4^+^T cell deleted murine pregnancy. Compared with Tim‐3^+^CD4^+^T cell transferred group, additional application with ML348 caused an increased vulnerability to fetal loss, characterized by a higher incidence of embryonic resorption and striking defect in the development of the labyrinth zone. Enhanced palmitoylation also impaired the tolerance phenotype of dCD4^+^T cells, as evidenced by the reduced production of IL‐4, IL‐10, IL‐13 and TGF‐β1 (Figure 7G). These data indicated that enhanced palmitoylation level of Tim‐3 eliminated the protective effect of Tim‐3^+^CD4^+^T cells on maternal‐fetal tolerance and fetal protection.

## 3 Discussion

**Figure 8:**
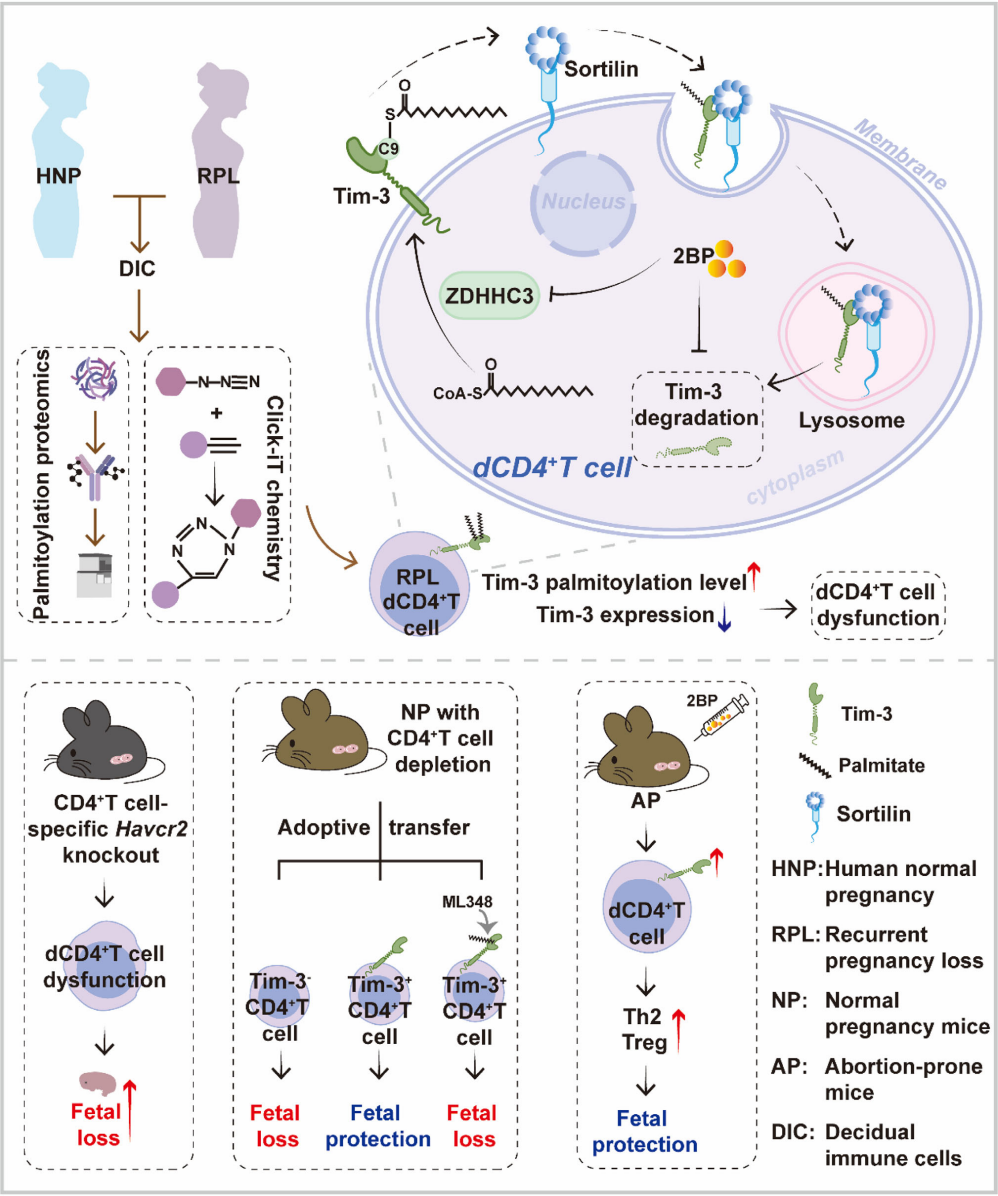
Schematic diagram of dysfunctional decidual CD4^+^T cells induced RPL via palmitoylation‐dependent Tim‐3 lysosomal sorting and degradation. Our integrated bioinformatics, proteomic, functional and model studies elucidated that Tim‐3 was palmitoylated by ZDHHC3 at Cys9. Sortilin bound to and directed palmitoylated Tim‐3 toward lysosome degradation, resulting in diminished Tim‐3 expression and the subsequent dysfunction of dCD4^+^T cells, which contributed to fetal loss. Inhibition of palmitoylation by 2BP increased Tim‐3 expression on dCD4^+^T cells, promoted dCD4^+^T cell tolerance and decreased the embryo resorption rate of AP mice. This schematic clarified a central role for dynamic palmitoylation in RPL, and uncovered a regulatory axis governing Tim‐3 homeostasis.

The psychological and physical burden imposed on women by RPL is often intolerable, with each successive loss exacerbating the burden. Nonetheless, advancements in prediction and prevention of RPL have been impeded by the enigmatic nature of its pathogenesis. The etiology of RPL is exceedingly intricate, with approximately 50% of cases lack well‐defined causative factors.^[^ ^3^, ^5^ ^]^ Empirical evidence suggests a correlation between maternal immune imbalance and the pathogenesis of RPL.^[^ ^7^, ^36^, ^37^ ^]^ In the present study, we uncovered a regulatory mechanism governing Tim‐3, which contributes to its decreased expression on dCD4^+^T cells in the context of miscarriage. We demonstrated that ZDHHC3‐mediated palmitoylation promoted lysosomal degradation of Tim‐3, leading to the dysfunction of dCD4^+^T cells and consequently, resulting in miscarriage (**Figure** **8**). Additionally, we have also identified that suppression of palmitoylation as an important potential therapeutic avenue for RPL.

Palmitoylation, a fully reversible post‐translational modification, regulates the properties and functions of a plethora of proteins. Although the dysregulation of palmitoylation has been recognized as a key mechanism for numerous pathological processes,^[^ ^20^, ^38^, ^39^ ^]^ little research addressed protein palmitoylation changes linked to RPL. By palmitoylation proteomics, we identified 3262 proteins with increased palmitoylation level and 1577 proteins with decreased palmitoylation level in DICs from RPL patients compared to HNP. Though the direct evidence that ML348 treatment increases fetal loss of NP mice is needed in the future, the general palmitoylation inhibitor, 2BP, mitigated the resorption rate of AP mice, further suggesting a correlation between elevated palmitoylation rate and RPL.

Previous studies highlight the significance of palmitoylation in regulating T cell responses. The palmitoylation cycle has been shown to be integral to several key cellular pathways in T cell responses, such as the STAT3 signaling pathway, PD‐L1 and interferon gamma receptor 1 degradation, and antigen cross‐presentation.^[^ ^19^, ^25^, ^40^, ^41^ ^]^ Reduced palmitoylation by 2BP also regulated dCD4^+^T cell functions. Palmitoylation proteomics identified increased palmitoylation of Tim‐3 on DICs during RPL. Tim‐3, initially identified as a molecule selectively expressed on terminally differentiated interferon‐γ‐secreting Th1 cells, is an important negative regulator of effector T cell responses in the context of tumors and infections.^[^ ^42^ ^]^ Dual blockade of Tim‐3 and CTLA‐4 (or PD‐1) pathways induces fetal loss with altered cytokine profiles by dCD4^+^T cells.^[^ ^43^, ^44^ ^]^ Tim‐3^+^Tregs also play a central role in the acceptance of the fetus during early stages of normal pregnancy,^[^ ^45^ ^]^ suggesting that Tim‐3 might be the key palmitoylated protein that regulates dCD4^+^T cell function during pregnancy.

Our bioinformatics and functional studies have revealed that ZDHHC3 palmitoylates Tim‐3 on Cys9 during pregnancy. Recently, Zhang et al. have documented that human Tim‐3 undergoes palmitoylation by ZDHHC9 at Cys296 residue.^[^ ^26^ ^]^ This divergence may stem from that Zhang et al.’s investigation centered on the distinct 11 additional amino acid residues (291 to 301) at the cytoplasmic tail of human Tim‐3, which are not present in the murine counterpart, while our research focused on the six cysteine residues that are evolutionarily conserved across the different species. In addition, in our quest to identify the ZDHHCs operative at the maternal‐fetal interface, we conducted a screen of those expressed in decidua, and ZDHHC9 had little expression in decidua based on the Human Protein Atlas. This discrepancy between the two studies further confirmed the palmitoylation of Tim‐3, and fully demonstrated that Tim‐3 could be palmitoylated at multiple cysteine residues, catalyzed by a variety of ZDHHC enzymes.

A multitude of evidence has underscored the pivotal role of Tim‐3 signaling in maternal‐fetal tolerance and trophoblast invasion, with its dysregulation being implicated in RPL.^[^ ^46^, ^47^, ^48^ ^]^ We provided direct insight into the role of Tim‐3 on dCD4^+^T cells and pregnancy outcome in vivo by CD4^+^T cell depletion and CD4^+^T cell‐specific Tim‐3 knockout mouse models. Despite this, the precise molecular mechanism for the decreased expression of Tim‐3 in RPL patients remain to be comprehensively elucidated. Our series of molecular and cellular experiments have unveiled the mechanism for palmitoylation‐dependent destabilization of Tim‐3. Palmitoylation of Tim‐3 by ZDHHC3, which facilitated its trafficking to the lysosome by sortilin, promoted the lysosomal degradation of Tim‐3. This degradation cascaded into Tim‐3 downregulation, dCD4^+^T cell dysfunction, and ultimately, fetal loss. Reduced palmitoylation by 2BP upregulated Tim‐3 expression on dCD4^+^T cells and diminished the embryo resorption rate of AP mice. Conversely, enhanced palmitoylation decreased Tim‐3 expression on dCD4^+^T cells, thereby eliminating the protective effect of Tim‐3^+^CD4^+^T cells on maternal‐fetal tolerance. Though the direct evidence that Cys9 mutation of Tim‐3 retains immune regulatory function in vivo by using point mutation mouse models is needed in the future, the effects of targeting Tim‐3 palmitoylation on Tim‐3 expression, dCD4^+^T cell function and pregnancy outcome underscore the potential of this post‐translational modification as a promising therapeutic strategy against RPL.

There are some limitations to our study that warrant further consideration. The use of broad‐spectrum palmitoylation inhibitor or promoter could also regulate other palmitoylated immune regulators. Though Cys9 mutation abolished Tim‐3 palmitoylation and stabilized the protein in vitro, we did not clarify whether mutation of Tim‐3 on Cys9 retains immune regulatory function in vivo. We observed higher expression levels of ZDHHC3 on dCD4^+^T cells from RPL patients than those from HNP, but the mechanisms underlying palmitoylation enhancement during RPL remain unclear. In addition, palmitoylation altered cytokine profiles by dCD4^+^T cells, but the specific roles and interrelationships of these cytokines at different stages of pregnancy have not yet been thoroughly explored. Such studies would be important for gaining insight into Tim‐3 palmitoylation and developing novel therapeutic approaches of RPL.

In conclusion, our research has delineated the pivotal role for dynamic palmitoylation in the context of RPL and identified a novel regulatory mechanism governing Tim‐3 expression, thereby expanding the current paradigm of Tim‐3 modulation during RPL. Furthermore, given the frequent association between Tim‐3 dysregulation and RPL, our findings may hold important implications for elucidating the etiology of RPL and for devising targeted therapeutic interventions for RPL. Targeting specific palmitoylases or depalmitoylases may represent a promising anti‐tumor therapeutic strategy, however, in conjunction with our present study, we believed that reproductive safety must be a paramount consideration when employing these strategies before or during pregnancy.

## 4 Experimental Section

### Ethical Approval

This study was approved by the Research Ethics Committee of the Obstetrics and Gynecology Hospital, Fudan University (No. Kyy2023‐82, 2023‐FCYY‐77JZS). Every participant signed a written informed consent form. All of animals were conducted in accordance with the National Guidelines for Animal Care and Use in Research (China). The experimental procedures were carried out in accordance with the approved guidelines.

### Human Samples

Decidual tissues of human first‐trimester pregnancies were obtained from clinically normal pregnancies (terminated for non‐medical reasons, with participants having at least one successful pregnancy and no history of spontaneous abortions, N = 61) and from miscarriages (specifically diagnosed as RPL, and excluding cases attributed to endocrine, anatomic, genetic abnormalities, infection, etc., N = 82). DICs were extracted through enzymatic digestion of decidual tissue in Roswell Park Memorial Institute (RPMI) 1640 medium (HyClone, U.S.A), supplemented with collagenase type IV (1.0 mg mL^−1^, CLS‐1, Worthington Biomedical, U.S.A) and DNase I (150 U mL^−1^, Applichem, Germany), following a previously established protocol.^[^ ^44^ ^]^ Subsequently, Tim‐3^+^CD4^+^T cells were isolated by magnetic affinity cell sorting using CD4 microbeads (MiltenyiBiotec, Germany) and then refined by BD FACSAria III Cell Sorter using PE‐conjugated anti‐human Tim‐3 antibody (Biolegend, U.S.A.).

### Cell Culture and Treatment

Freshly isolated dCD4^+^T cells were cultured at a density of 5 × 10^5^ cells per well and subjected to a range of concentrations of 2BP (Sigma‐Aldrich, USA, 0, 10, 25, 50, 100 µM), ML348(MCE, USA, 0, 1, 5, 10, 20 µM) for a duration of 48 h, or alternatively treated with 50 µM 2BP, 10 µM ML348 for periods of 0 h, 24 h, 48 h, 72 h. In some experiments, MG132 (MCE, USA, 1 µM), 3MA (MCE, USA, 10 mM) or CQ (Sigma‐Aldrich, 20 µM) was added into the treatment regimen. Phorbol 12‐myrstate 13‐acetate (PMA) (50 ngmL^−1^, Biolegend, U.S.A.), ionomycin (1 µg mL^−1^, Biolegend, U.S.A.), brefeldin A (10 mg mL^−1^, BioLegend, U.S.A.) were introduced 4 h before the end of the 48 h co‐culture period.

HEK293T (ATCC, CRL‐11268) cells were cultured in Dulbecco's Modified Eagle Medium‐high glucose (DMEM‐high glucose, Hyclone)‐enriched with 10% fetal bovine serum (FBS, Gibco), 100 U mL^−1^ penicillin and 100 µg mL^−1^ streptomycin unless otherwise indicated. All cell cultures were incubated at 37^ °^C in an atmosphere with 5% CO_2_.

Jurkat T (Clone E6‐1, ATCC, TIB‐152) cells were cultured in RPMI 1640 medium ‐enriched with 10% FBS, 100 U mL^−1^ penicillin and 100 µg mL^−1^ streptomycin unless otherwise indicated. All cell cultures were incubated at 37^ °^C in an atmosphere with 5% CO_2_.

### Cell Transfection

To construct *Tim ‐ 3 ‐ WT*, *Tim ‐ 3 ‐ C9A* and *ZDHHC3* overexpression plasmids (oe‐*Tim ‐ 3 ‐ WT*, oe‐*Tim ‐ 3 ‐ C9A‐Flag*, oe‐*ZDHHC3‐Myc*), the respective sequence of *Tim ‐ 3 ‐ WT*, *Tim ‐ 3 ‐ C9A* and *ZDHHC3* were amplified and cloned into the vector (Public Protein/Plasmid Library, PPL, China). Specific knockdown of *ZDHHC3, ZDHHC5, ZDHHC7, ZDHHC17, ZDHHC18, ZDHHC24* and *SORT1* was achieved by siRNA (si‐*ZDHHC3*, si‐*ZDHHC5*, si‐*ZDHHC7*, si‐*ZDHHC17*, si‐*ZDHHC18*, si‐*ZDHHC24*, si‐*SORT1*, Generay biotechnology, Shanghai, China, Table S1, Supporting Information). HEK293T cells were transfected with oe‐*Tim ‐ 3 ‐ WT*, oe‐*Tim ‐ 3 ‐ C9A‐Flag* and oe‐*ZDHHC3‐Myc* for 48 h using Polyethylenimine Linear (PEI) MW40000 (49553‐93‐7, Yeasen Biotechnology, Shanghai, China). Conversely, HEK293T cells were transfected with si‐*ZDHHC3*, si‐*ZDHHC5*, si‐*ZDHHC7*, si‐*ZDHHC17*, si‐*ZDHHC18*, si‐*ZDHHC24* and si‐*SORT1* for 48 h using transfection reagent (L3000015, Thermo Fisher Scientific, U.S.A). Jurkat T cells were transduced with lentivirus carrying the indicated plasmid.

### Palmitoylation Proteomics

Palmitoylation‐enriched samples were first obtained from DIC of HNP and RPL patients. Following tryptic digestion, peptides were desalted utilizing C18 spin column (Thermo Scientific). Post‐drying, peptides were resuspended in 200 µL loading buffer and mixed with 100 µL of high‐capacity streptavidin beads. The eluted peptides were then combined with 50 mM iodoacetamide to block reduced cysteine residues, which indicated the palmitoylation sites. Finally, the peptides subsequently subjected to further desalting with C18 StageTips and in preparation for LC‐MS/MS analysis. LC‐MS/MS were performed on an Orbitrap Astral mass spectrometer coupled with Vanquish Neo UHPLC system (Thermo Fisher Scientific) in Climb Technology Co., Ltd. The data were analyzed using Spectronaut 18 software (Biognosys AG, Switzerland) and searched against the UniProtKB reviewed (Swiss‐prot) database. Peptide matches were filtered at 1% FDR. Site quantitation analysis was filtered only for those carbamidomethylated cysteine sites that were confidently localized ≥0.75 site probability with algorithm.

### Click‐iT Identification of Tim‐3 Palmitoylation

After 48 h post‐transfection with oe‐*Tim ‐ 3 ‐WT* (followed by transfection with or without si‐*ZDHHC3*) or oe‐*Tim ‐ 3 ‐ C9A*, 100 µM of Click‐iT palmitic acid‐azide was added to the cell medium with gentle mixing, followed by a 6 h incubation at 37 °C under 5% CO_2_ atmosphere. Upon completion of the incubation period the medium was removed and the cells were extensively rinsed three times with phosphate buffered saline (PBS) before the addition of lysis buffer (1% sodium dodecyl sulfate in 50 mM Tris‐HCl, pH 8.0) containing protease inhibitor and phosphatase inhibitor at appropriate concentrations. Cell lysate was incubated for 20 min on ice and transferred into a 1.5 mL microcentrifuge tube. Then, the lysate was sonicated with a probe sonicator to solubilize the proteins and disperse DNA before centrifugation at 12 000 rpm at 4 °C for 20 min. The supernatant was transferred to a fresh tube and the protein concentration was quantified by BCA Protein Assay Kit (WB6501, ncmbiotech, China). Whereafter, the protein sample was reacted with biotin‐alkyne using the Click‐iT Protein Reaction Buffer Kit (catalogue number C10276; Thermo Fisher Scientific) following the protocols from the instruction sheet. Biotin alkyne‐azide‐plamitic‐protein complex was subsequently pulled down by streptavidin magnetic beads, with the reacted protein lysates and streptavidin magnetic beads incubating overnight at 4 °C. After thorough washing, protein loading buffer was added to the magnetic beads bound with the protein complex before heating at 100 °C for 10 min. The resulting protein samples were finally subjected to immunoblotting detection for Tim‐3 expression.

### Palmitoylated Protein Assay

Palmitoylated proteins were detected according to the protocol of the Palmitoylated Protein Assay Kit (ab273279, Abcam, USA). Briefly, the cells were plated in a 96‐well cell culture plate in RPMI 1640 Medium with 1× Palmitic Acid Label for 24 h. Then the cells were collected and washed by PBS before fixation and permeabilization. Subsequently, palmitic acid reaction was conducted according to the protocols from the instruction sheet. Once the reaction was complete, the cells were analyzed using flow cytometry analysis.

### Co‐Immunoprecipitation

HEK293T cells were transfected with either *Tim ‐ 3 ‐ WT* or *Tim ‐ 3 ‐ C9A* plasmid for a period of 48 h. In certain experiments, transfection with si‐*ZDHHC3*, 2BP or ML348 were also included. Subsequently, cells were washed with cold PBS and lysed using a lysis buffer (Beyotime, 0013J) containing protease inhibitor. After a 20‐min incubation on ice and gentle sonication, the lysates were collected into a 1.5 mL microcentrifuge tube and centrifuged at 12 000 rpm at 4 °C for 20 min. The supernatant was then transferred to a clean tube and protein concentration was determined using BCA Protein Assay Kit (WB6501, ncmbiotech, China). Whereafter, for IP, each protein sample, adjusted to a concentration of 1 mg in 300 µL lysis buffer, were incubated with protein A/G MagBeads (36417ES03, Yeasen Biotechnology (Shanghai) Co., Ltd.) overnight at 4 °C. Notably, the protein A/G MagBeads had been pre‐incubated with Tim‐3 antibody. After thorough washing, protein loading buffer was added to the MagBeads bound with the protein complex, and the mixture was heated at 100 °C for 10 min. The protein samples were then ready for immunoblotting detection.

### Western Blot

Western blot was performed in accordance with established methods.^[^ ^49^ ^]^ The cell samples were lysed with cold radio‐immunoprecipitation (RIPA) buffer (Beyotime Biotechnology, Shanghai, China) supplemented with a protease inhibitor cocktail (MCE, Shanghai, China) and a phosphatase inhibitor (TargetMol, China). Protein concentration was determined by BCA Protein Assay Kit (WB6501, ncmbiotech, China). Aliquots of 20 µg protein from each sample was denatured at 100 °C for 10 min and subjected to electrophoresed in 7.5% SDS‐polyacrylamide gels (P2011, ncmbiotech, China). Subsequently, the proteins were transferred to 0.45 µm polyvinylidene fluoride (PVDF) membranes (Millipore, Germany). Non‐specific binding was blocked with 5% skimmed milk (Servicebio, China) and membranes were incubated overnight at 4 °C with the following primary antibodies: anti‐Tim‐3 (ab241332, Abcam), anti‐sortilin (12369‐1‐AP, Proteintech), anti‐GODZ (ZDHHC3) (DF13476, Affinity), anti‐LGMN (A23776, abclonal). β‐Tubulin (ab6046, Abcam) and β‐Actin (ab179467, Abcam) served as internal standards. Membranes were washed and incubated with horseradish peroxidase (HRP) conjugated secondary antibody (Jackson, U.S.A) at room temperature for 1 h. Chemiluminescence was employed to detect antibody‐labeled proteins (Amersham Imager 600, GE Healthcare, U.S.A) using Chemiluminescent Kit (Share‐bio, China).

### CHX Chase Assay

HEK293T cells were transfected with *Tim ‐ 3 ‐ WT* or *Tim ‐ 3 ‐ C9A* plasmid for 48 h. In certain experiments, cells were co‐transfected with si‐*ZDHHC3*, treated with 50 µM 2BP or 10 µM ML348. Prior to cell sample collection, the culture medium was replaced with medium fresh containing CHX (50 µg mL^−1^), and the cells were further incubated at 37 °C for specified time intervals. The alterations in Tim‐3 protein levels were quantified by immunoblot.

### Mass Spectrometric Analysis

Equal amounts of proteins with or without 50 µM 2BP treatment were treated with 25 mM dithiothreitol (DTT) into the above protein solution to achieve a final concentration of approximately 5 mM. An enzymolysis diluent was added to redissolve the protein precipitate, ensuring a protein‐to‐enzyme ratio of 50:1 (g/g) Tandem Mass Tag (TMT) labelling was then performed. The labeled samples were subsequently subjected to the LC‐MS analysis by Shanghai Luming biological technology co., LTD (Shanghai, China). Proteome Discoverer (v.2.4) was used to search the raw data thoroughly against the Uniprot Homo sapiens database. Fold change (>1.5 or <0.66), along with a P‐value < 0.05 were used to considered significantly differentially expressed.

### Mice

Female CBA/J mice, male DBA/2 mice, and male BALB/c mice were sourced from Slac laboratory animal Co. (Shanghai, China) and Huafukang bioscience Co. (Beijing, China) and housed in an accredited animal facility, adhering to institutional and National Institutes of Health Guidelines. Eight‐week‐old CBA/J females were mated with BALB/c males to induce NP models. Eight‐week‐old CBA/J females were mated with DBA/2 males to establish AP models. All the CBA/J females were inspected every morning to detect the presence of vaginal plugs. The day of visualization of a plug was designated as day 0.5 of gestation (GD 0.5).

Some of the AP pregnant mice received an intraperitoneal injection of 2BP at a frequency of three times per week. Specifically, 0.032 g 2BP powders were dissolved in 2 mL of DMSO following to add 8 mL of PEG300 (targetmol, T7022), 1 mL of Tween‐80 (targetmol, T13947), and 9 mL of physiological saline. Mix thoroughly until a clear, transparent solution is obtained. Intraperitoneally injection at a dose of 200 µL per 10 g of body weight. And prepare DMSO vehicle solution (same composition without 2BP) for control group.

For CD4^+^T cell depletion and CD4^+^T cell adoptive transfer in pregnant CBA/J mice, anti‐mouse CD4 mAb (GK1.5, Biolegend) were injected intraperitoneally at GD1.5 (200 µg) and GD4.5 (100 µg), in accordance with previous publications.^[^ ^50^, ^51^ ^]^ Tim‐3^+^CD4^+^T or Tim‐3^−^CD4^+^T cells were isolated from the spleens of NP CBA/J mice by BD FACSAria III Cell Sorter. The sorted cells were resuspended in 200 µL of PBS and injected into the tail vein of CD4^+^T cell‐depleted pregnant mice at GD5.5. In certain experimental groups, Tim‐3^+^CD4^+^T cells were pre‐stimulated with ML348 (10 µM, MCE, USA) for 48 h prior to adoptive transfer.

All knockout mice were genetically engineered by Cyagen Biotechnology Co., Ltd (Suzhou, China). For the generation of the *HAVCR2* conditional knockout group, female *Cd4^Cre^* ^+/−^ *Havcr2* ^fl/fl^ mice were mated with *Cd4^Cre^* ^−/−^ *Havcr2* ^fl/fl^ male mice. For the control group, Female *Cd4^Cre^* ^−/−^ *Havcr2* ^fl/fl^ mice were mated with *Cd4^Cre^* ^+/−^ *Havcr2* ^fl/fl^ male mice. All pregnant mice were monitored at GD13.5. The percentage of fetal loss (the embryo absorption rate) was calculated using the following formula: % of resorption = R/(R+V) ×100, where R represents the number of hemorrhagic implantation sites (sites of fetal loss) and V stands for the number of viable, surviving fetuses.

Uteri from pregnant mice were dissected to remove the mesometrium and were excised at the ovaries and cervix. The fetal and placental tissues were carefully removed and rinsed in PBS. Minced uteri were digested in RPMI 1640, supplemented with collagenase type IV and DNase I for 45 min at 37 °C with gentle agitation. The resulting cells were cultured in RPMI 1640 enriched with 10% FBS, 100U/mL penicillin, 100 µg mL^−1^ streptomycin, and 1 µg mL^−1^ amphotericin B at 37 °C in an atmosphere of 5% CO_2_ for 4 h, allowing for the selective detachment of adherent stromal cells. The spleen was aseptically excised and immediately stored in RPMI 1640. To obtain a single‐cell suspension, the spleen tissue was mechanically disrupted using a 10‐mL syringe plunger to pass it through a 70‐µm mesh strainer. PMA (50 ng mL^−1^, Biolegend, U.S.A.), ionomycin (1 µg mL^−1^, Biolegend, U.S.A.) brefeldin A (10 mg mL^−1^, BioLegend, U.S.A.), were added to the cell cultures and incubated for 4 h for intracellular cytokine analysis of T cells.

### Immunofluorescence and Hematoxylin‐Eosin (HE) Staining

Paraffin‐embedded sections of tissues were dewaxed using dimethylbenzene and subsequently rehydrated through a graded ethanol series (100%, 95%, 90%, 80%, 70% and 50%). For paraffin section immunofluorescence, the sections were first blocked with 10% donkey serum (SL050, solarbio, China) after antigen retrieval using citric acid antigen repair buffer (pH 6.0, G1219‐1L, Servicebio, China). For cell crawling sheet immunofluorescence, HEK293T cells, cultured to optimal density, were transfected with *Tim ‐ 3 ‐ WT* plasmid and treated with 50 µM 2BP or 10 µM ML348. The slides of cells were washed three times in PBS, fixed with 4% paraformaldehyde (G1101, Servicebio, Wuhan, China), permeabilized with 0.5% Triton X‐100 (T8200, Solarbio, China) and blocked with 10% donkey serum (SL050, solarbio, China). And they were then incubated with a panel of primary antibodies: Tim‐3/HAVCR2 Rabbit pAb (A13443, abclonal), anti‐Human CD107a/LAMP1 (H4A3) (65051‐1‐Ig, Proteintech), Tim‐3 monoclonal antibody (60355‐1‐Ig, Proteintech), sortilin polyclonal antibody (12369‐1‐AP, Proteintech), LAMP1/CD107a Rabbit PolymAb (A24804, abclonal), GODZ (DHHC3) Antibody (DF13476, Affinity), human CD4 Antibody (AF‐379‐NA, R&D) overnight at 4 °C. On the following day, the sections were incubated with Alexa Fluor‐conjugated secondary antibodies (AS027, AS039, AS053, AS054, ABclonal, China) for 1.5 h at room temperature, followed by 4′,6‐diamidino‐2‐phenylindole (DAPI) staining. After washed three times with tris‐buffered saline (TBS) for 10 min each, the sections were sealed with anti‐fade mounting medium to preserve fluorescence. Images were captured on a THUNDER Imaging Systems (Leica, Germany).

For HE staining, the sections were stained with hematoxylin solution for 5 min, and, followed by a brief rinse in ultrafiltration water for 5 sec. Next, the sections were stained with eosin solution for 3 min and dehydrated through an ethanol gradient (50%, 70%, 80%, 90%, 95% and 100%) and cleared in dimethylbenzene. The slides were sealed with mounting medium and taken pictures using a bright‐field microscope (Olympus, Japan). The area of labyrinthine zone within each analyzed image was calculated by ImageJ software.

### Flow Cytometry

Cell surface molecular expression and intracellular cytokine production were evaluated using flow cytometry. A panel of fluorochrome‐conjugated antibodies was employed to detect specific markers: FITC‐conjugated anti‐mouse IL‐4, IL‐10, anti‐human CD14; PE‐conjugated anti‐human Tim‐3, anti‐mouse Tim‐3, IL‐13; PE/CY7‐conjugated anti‐human IL‐10, TGF‐β1, CD56; PerCP/Cy5.5‐conjugated anti‐human IL‐4, CD11c; Alexa Fluor 647‐conjugated anti‐human IL‐13; Brilliant Violet 421‐conjugated anti‐human IL‐4, CD8, anti‐mouse TGF‐β1; Brilliant Violet 510‐conjugated anti‐human CD4, anti‐mouse CD4; Brilliant Violet 605‐conjugated anti‐human CD4; APC/CY7‐conjugated anti‐human CD3 (Biolegend, U.S.A.) antibodies. For intracellular staining, cells were fixed and permeabilized using the Fix/Perm kit (Biolegend, U.S.A.). Flow cytometry was performed on a Beckman‐Coulter CyAn ADP cytometer (Beckman‐Coulter, U.S.A.) and analyzed with FlowJo software (Tree Star, Ashland, U.S.A.).

### Statistical Analysis

Data were tested for normal distribution (Kolmogorov‐Smirnov), defining whether the results should be analyzed parametrically or non‐parametrically. For the normally distributed data, significance of differences between two groups was determined by Student's t‐test. For the non‐normally distributed data, significance of differences between two groups was determined by Mann‐Whitney‐test. Multiple groups were analyzed by one‐way ANOVA with the post‐hoc Dunnett t‐test using Prism Version 8 software (GraphPad, San Diego, CA, USA). Variables were presented as means and standard error of mean (SEM). For all statistical tests, *p*‐values < 0.05 were considered statistically significant.

## Conflict of Interest

The authors declare no conflict of interest.

## Author Contributions

L.C. and X.M. contributed equally to this work. S.W., M.Q. and L.C. initiated and supervised the project. L.C., S.W. and X.M. carried out experiments and analyzed data. M.Q., J.Q., X.M. and Y.L. coordinated the sample collection, data interpretation, literature search, and Figure preparation. S.W. drafted the manuscript. L.C., F.S., M.Q., and X.M. revised the manuscript. All authors approved the final manuscript.

## Supporting information



Supporting Information

## Data Availability

The data that support the findings of this study are available from the corresponding author upon reasonable request.
