# Supplementary material for: Dysfunctional Decidual CD4+T Cells Induce Recurrent Pregnancy Loss via Palmitoylation‐Dependent Tim‐3 Lysosomal Sorting and Degradation
Source: Adv Sci (Weinh). 2025 Jul 11;12(38):e00971. doi: 10.1002/advs.202500971 (PMC12520500; doi:10.1002/advs.202500971)
Supplement: Supplementary file 1 — Supporting Information [file ADVS-12-e00971-s001.docx]

**Supporting Information**

**Dysfunctional decidual CD4^+^T cells induce recurrent pregnancy loss via palmitoylation-dependent Tim-3 lysosomal sorting and degradation**

*Liyuan Cui, Xinhang Meng, Yujie Luo, Jinfeng Qian, Fengrun Sun, Mingke Qiu*, Songcun Wang**

The Supplementary Materials include:

Figure S1-S5

Table S1


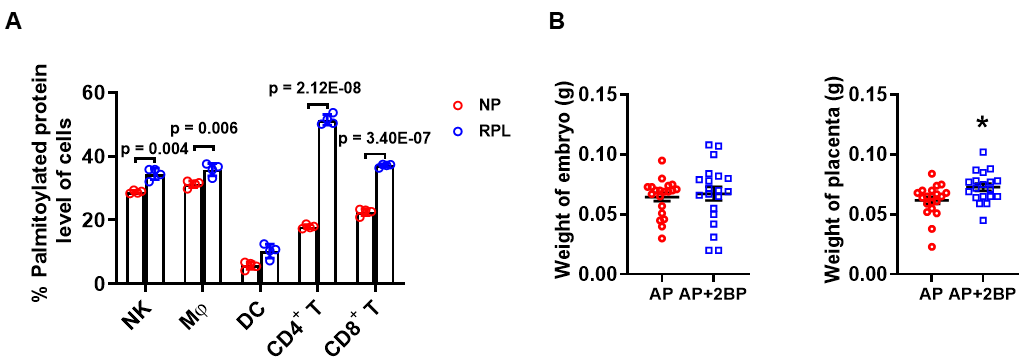


**Figure S1. A** Comparative palmitoylated protein assay of different types of DICs (NK cells, macrophages (Mφ), Dendritic cells (DC), CD4^+^T cells, CD8^+^T cells) between HNP (n=4) and RPL (n=4). **B** The weight of embryos and placentae of AP mice with or without 2BP treatment.


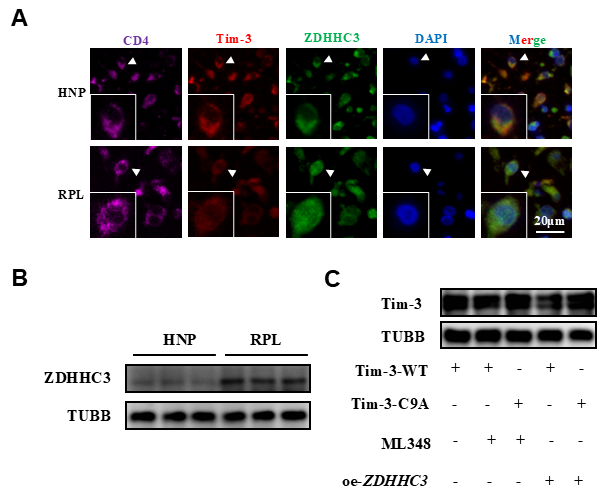


**Figure S2. A** Fluorescent colocalization of Tim-3 and ZDHHC3 in dCD4^+^T cells from paraffin sections of decidual tissue of HNP and RPL. Images are representative of three individual experiments. **B** Protein levels of ZDHHC3 in dCD4^+^T cells from HNP and RPL were examined by Western blot. β-tubulin (TUBB) was used as an internal control. **C** Protein levels of Tim-3 in Jurkat T cells with indicated treatment were examined by Western blot. TUBB was used as an internal control. Images are representative of three individual experiments.


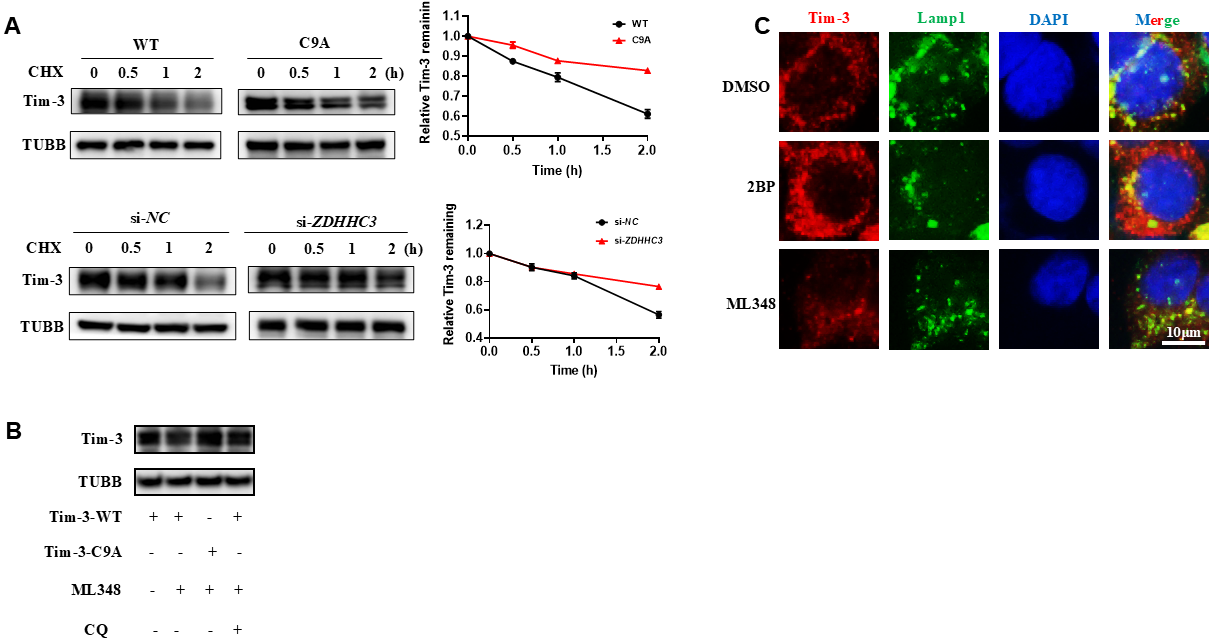


**Figure S3. A** Upper: Half-life of Tim-3, Tim-3-C9A mutant (Tim-3-C9A) measured by CHX chase assay. Lower: Tim-3 half-life in si-*NC*/si-*ZDHHC3* transfected HEK293T cells measured by CHX chase assay. Relative protein levels of Tim-3 normalized to TUBB protein were presented relative to the level at 0 h after CHX treatment. **B** Protein levels of Tim-3 in HEK293T cells with indicated treatment were examined by Western blot. TUBB was used as an internal control. **C** Representative images for the colocalization of Tim-3 and Lamp1 (a marker for lysosome) in HEK293T cells treated with 50 μM 2BP or 10 μM ML348. Images are representative of three individual experiments.


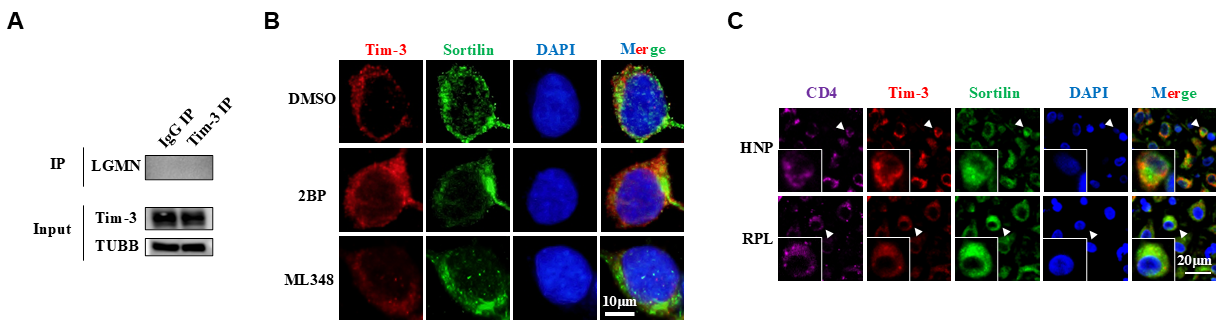


**Figure S4****. The interaction of Tim-3 and LGMN or Sortilin. A** IP analysis demonstrating the interaction of Tim-3 and LGMN. **B** Representative images for the colocalization of Tim-3 and Sortilin in HEK293T cells treated with 50 μM 2BP or 10 μM ML348. **C** Representative images for the colocalization of Tim-3 and Sortilin in dCD4^+^T cells from paraffin sections of decidual tissue of HNP and RPL. Images are representative of three individual experiments.


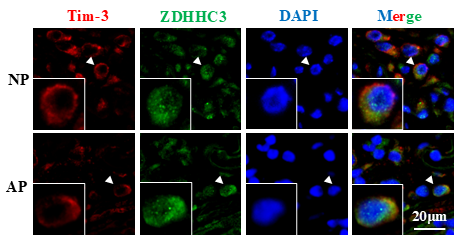


**Figure S5.** Representative images for the colocalization of Tim-3 and ZDHHC3 at the maternal-fetal interface from NP and AP. Images are representative of three individual experiments.

**Table S1. siRNA list.**

| **Name of siRNA** | **Sense (5'-3')** | **Antisense (5’-3’)** |
| --- | --- | --- |
| *si- homo ZDHHC3* | CGUUCUCAUGAAUGUUUAATT | UUAAACAUUCAUGAGAACGTT |
| *si- homo ZDHHC5* | GAAAGAGAAGACAAUUGUATT | UACAAUUGUCUUCUCUUUCTT |
| *si- homo ZDHHC7* | CAAGUGAUGUUUAGAAAUATT | UAUUUCUAAACAUCACUUGTT |
| *si- homo ZDHHC17* | GGAUGUGUAAGAAAUAUUATT | UAAUAUUUCUUACACAUCCTT |
| *si-homo ZDHHC18* | GGCAGAUGGUGAAGCUGAATT | UUCAGCUUCACCAUCUGCCTT |
| *si- homo ZDHHC24* | AGAACAAGAUUGUCUCAAATT | UUUGAGACAAUCUUGUUCUTT |
| *si-homo SORT1①* | GAGAACUCUGGAAAGGUGGUGUUAA | UUAACACCACCUUUCCAGAGUUCUC |
| *si-homo SORT1②* | CCUACAGCAUCUCCCAGAAACUGAA | UUCAGUUUCUGGGAGAUGCUGUAGG |
| *si-homo SORT1③* | CAGGAGUGCUCAUUGUGAAGAAAUA | UAUUUCUUCACAAUGAGCACUCCUG |
